# Effects of different feeding patterns on the rumen bacterial community of tan lambs, based on high-throughput sequencing of 16S rRNA amplicons

**DOI:** 10.3389/fmicb.2023.1228935

**Published:** 2023-10-19

**Authors:** Lili Zhang, Wenyi Ren, Yanliang Bi, Jie Zhang, Yuchen Cheng, Xiaofeng Xu

**Affiliations:** ^1^College of Animal Science and Technology, Ningxia University, Yinchuan, China; ^2^Institute of Feed Research, Chinese Academy of Agricultural Sciences, Beijing, China

**Keywords:** alpha diversity, beta diversity, fatty acids, pasture-fed, pen-fed, tan lambs

## Abstract

**Introduction:**

The mutton quality of Chinese Tan lambs (*Ovis aries*) has declined as feeding patterns have shifted from pasturing to pen rationing. While pen-fed can enhance the growth performance of sheep, it falls short in terms of meat quality attributes such as meat color and tenderness. Furthermore, compared to pen-fed, pasture-fed husbandry increases the proportion of oxidative muscle fibers, decreases the proportion of glycolytic muscle fibers, and reduces LDH (lactate dehydrogenase) activity. Mutton quality is affected by fatty acids, and rumen microorganisms play a role in the synthesis of short-chain fatty acids, long-chain fatty acids, and conjugated linoleic acids.

**Methods:**

We used 16S rRNA sequencing to analyze the effects of two feeding patterns on the rumen bacteria of Tan lambs. In a randomized block design with 24 newborn Tan lambs, 12 lambs were fed by ewes in pasture and 12 were fed by pen-fed ewes. At 2 months, the biceps femoris and the longissimus dorsi were analyzed by gas chromatography for intramuscular fat content and fatty acids composition, and DNA in the rumen contents was extracted and used to analyze the structure of the bacterial community.

**Results:**

Different feeding patterns had no significant effect on the intramuscular fat content of the biceps femoris and longissimus dorsi of the lambs, but there was a significant effect on fatty acids composition. The fatty acids c18:3n3 and c20:5n3 were significantly higher in the biceps femoris and longissimus dorsi of the pasture group than the pen-ration group. The alpha diversity of rumen bacteria was significantly greater in the pasture group compared to the pen-ration group. The ACE index, Chao1 index, Shannon index, and Simpson index were all notably higher in the pasture group than in the pen-ration group. Utilizing beta diversity analysis to examine the differences in rumen bacteria between the pasture group and pen-ration group, it was observed that the homogeneity of bacteria in the pasture group was lower than that in the pen-ration group. Furthermore, the diversity of rumen bacteria in the pasture group was greater than that in the pen-ration group. Twenty-one phyla were identified in the pasture group, and 14 phyla were identified in the pen-ration group. The dominant phyla in the pasture group were Bacteroidetes and Fibrobacteres; the dominant phyla in the pen-ration group were Proteobacteria and Bacteroidetes. The relative abundance of Proteobacteria was significantly higher in the pen-ration group than in the pasture group (*p* < 0.01). Diversity at the genus level was also higher in the pasture group, with 176 genera in the pasture group and 113 genera in the pen-ration group. The dominant genera in the pasture group were *Prevotella_1*, *Rikenellaceae_RC9_gut_group*, and *Bacteroidales_BS11_gut_group_Na*; the dominant genera in the pen-ration group were *Prevotella_1*, *Prevotella_7*, *Succinivibrionaceae_UCG-001*, and *Succinivibrionaceae_NA*.

**Discussion:**

The rumen bacterial community of Tan sheep is significantly influenced by pen-ration and pasture-fed conditions, leading to variations in fatty acid content in the muscle, which in turn affects the flavor and nutritional value of the meat to some extent. Pasture-fed conditions have been shown to enhance the diversity of rumen bacterial community structure in Tan sheep, thereby increasing the nutritional value of their meat.

## 1. Introduction

The rumen is a complex microbial ecosystem that plays a vital role in digestion and the maintenance of health in ruminants (Zened et al., 2013). Many substances that cannot be used directly by ruminants are converted into accessible nutrients through fermentation in the rumen. At birth, the ruminant is free of bacteria, fungi, protozoa, and other microorganisms. Diet and age simultaneously drive changes in the structure and abundance of bacterial communities in the developing rumen (Juliana et al., 2017). With the intake of milk and contact with the environment, microorganisms begin to appear in the rumen and then multiply (Xiao et al., 2018). The development of the structure and function of the rumen microbial community in juveniles directly affects the production of adults. Moreover, Barbieri et al. (2015) found that the rumen microorganisms of lambs can be changed through diet (suckling) or inoculation of the rumen, and resulting changes in the rumen bacterial community can persist until weaning. Overall, rumen microorganisms play a substantial role in ruminant growth and fattening.

Fatty acids metabolism in the rumen has a major influence on the fatty acids composition of ruminant meats and milk and also affects the synthesis of flavor substances (Jenkins et al., 2007; Wang et al., 2019). The short-chain fatty acids produced and biohydrogenation by rumen microbial metabolism affect the transformation of polyunsaturated fatty acids to saturated fatty acids and the generation of conjugated fatty acids (Bessa et al., 2000; Jenkins et al., 2007). Conjugated fatty acids play a role in the deposition of intramuscular fat, reducing body fat and improving meat quality (Yang et al., 2019). In ruminant animals, intramuscular fat (IMF) is composed of approximately 45 to 48% saturated fatty acids (SFA), 35 to 45% monounsaturated fatty acids (MUFA), and around 5% polyunsaturated fatty acids (PUFA). The muscle tissue mainly contains medium-chain and long-chain fatty acids (c12 to c22), with a lower concentration of short-chain fatty acids (c4 to c10). The composition of fatty acids (FAs) in IMF is influenced by factors such as diet type, digestive system functionality, and the FA synthesis process within the animal’s body (Aldai et al., 2007; Dugan et al., 2007). Due to the hydrogenation activity of rumen microbes in ruminant animals, SFA content in ruminant IMF is higher than in monogastric animals, and the ratio of PUFA to SFA (P/S) is lower in ruminant animals compared to monogastric animals. The primary fatty acids in SFA are c16:0 and c18:0, while in MUFA, c18:1n-9 constitutes about 80% of total fatty acids. In PUFA, the main components are linoleic acid (18,2n-6) and alpha-linolenic acid (18,3n-3), which together make up around 2% of total fatty acids. Recent research has found a significant negative correlation between the content of alpha-linolenic acid (c18:3, LNA) in lamb meat and the relative abundance of the Prevotella genus, suggesting that Prevotella may be a key microbe involved in the hydrogenation of LNA in the rumen (Wang et al., 2018). However, there have been no studies reporting the correlation between the relative abundance of Prevotella and intermediate or end products of LNA rumen hydrogenation. Therefore, the exact contribution of Prevotella in the rumen hydrogenation process remains unknown. Additionally, strains such as *Megasphaera elsdenii*, Bifidobacteria, Lactobacilli, and Streptococci have been confirmed to participate in the rumen fatty acid hydrogenation process (Ando et al., 2004; Toral et al., 2012; Li et al., 2013). Boeckaert et al. (2008) also discovered a spiral-shaped bacterium that falls between the Butyrivibrio and Pseudobutyrivibrio genera and confirmed its involvement in the production of stearic acid (SA) during rumen hydrogenation. Different microbial species have varying abilities to hydrogenate polyunsaturated fatty acids. To date, *Butyrivibrio fibrisolvens* has been identified as one of the most active rumen bacteria in isomerizing linoleic acid (LA), secreting isomerase enzymes that convert LA into cis-9, trans-11 conjugated linoleic acid (CLA). Some strains, such as the cellulolytic *Butyrivibrio fibrisolvens* JW11, can further secrete reductase enzymes to convert cis-9, trans-11 CLA into trans-11-c18:1 TVA (trans-vaccenic acid). Microorganisms have a significant impact on the quality and fatty acid composition of ruminant meat. Research indicates that healthier ruminant products can be achieved through the effective manipulation of the rumen microbiota, aiming to reduce the final step of cis-9, trans-11 c18:2 biohydrogenation and increase the bypass of c18:3n-3, trans-11 c18:1, and cis-9, trans-11 c18:2 flux to the muscle (Chilliard et al., 2007; Toral et al., 2018). However, comparative studies on the association between rumen microbial diversity in Tan sheep under pasture and pen-ration conditions and the beneficial fatty acid content in their meat have not been reported.

We conducted a study to investigate the similarities and differences in 2-month-old Tan lambs raised under different feeding patterns, aiming to explore the effect of feeding patterns on the rumen microbiota and fatty acid composition of Tan lambs. This research provides a theoretical basis for understanding the relationship between the rumen microbiota of Tan sheep and the fatty acid composition in their meat, offering new insights into microbial interventions for improving meat quality.

## 2. Materials and methods

### 2.1. Experimental design

In this randomized block design, 24 healthy newborn Tan lambs (3.19 ± 0.21 kg, half male and half female) were divided into two feeding groups: a pasture group and a pen-ration group. The lambs in the pasture group followed the grazing lactating ewe to suckle and could freely eat the pasture grass. The composition of the pasture grass was 40% *Astragalus adsurgens*, 20% *Lespedeza davurica*, 5% *Sophora alopecuroides*, 10% *Caragana korshinskii*, 10% *Glycyrrhizae radix*, and 10% *Achnatherum splendens*. Lambs in the pen-ration group were fed with lactating ewes in the stable and were free to eat the starter at 3 weeks old. The composition of the starter feed is shown in Supplementary Table 1.

### 2.2. Sample collection

At the age of 2 months, five Tan lambs in each group were randomly selected and slaughtered. The rumen was removed immediately after slaughter, and 50 mL of its contents was collected and mixed. Then 10 g biceps femoris and 10 g longissimus dorsi were cut with surgical scissors 45 min after slaughter. All samples were put into a cryopreservation tube. The muscle samples were sent to the laboratory, preserved in liquid nitrogen, and stored at −80°C.

The rumen contents were transferred to a centrifuge bottle containing CO_2_ to maintain anaerobic conditions and kept on ice for no more than 20 min prior to processing. Cooling samples with ice does not affect the sample or subsequent analysis (Wu et al., 2010). The rumen contents were centrifuged at 10,000 g, and the precipitate was dissolved in extraction buffer (100 mM Tris–HCl, 10 mM ethylenediaminetetraacetic acid, 0.15 M NaCl, pH 8.0). A total of 1 g precipitate was dissolved in 4 mL buffer and incubated with the rumen contents at 4°C for 1 h to maximize the release of particle-associated bacteria by cooling (Dehority and Grubb, 1980). The suspension was centrifuged at 500 g for 15 min at 4°C to remove broken plant particles while keeping the bacterial cells suspended, then passed through a four-layer cheesecloth, centrifuged (10,000 g, 25 min, 4°C), and stored at −20°C until DNA extraction.

### 2.3. DNA extraction

We extracted DNA from pretreated rumen contents using the E.Z.N.A. stool DNA Kit (Omega Biotek, Norcross, GA, United States) according to the manufacturer’s protocols and stored it at −20°C. The quantity and quality of the DNA was measured with NanoDrop 2000 (Thermo Fisher Scientific, Wilmington, Del., United States).

### 2.4. Methods

#### 2.4.1. Determination of fat and fatty acids

Fat was extracted from the samples with the chloroform-methanol method (Folch et al., 1957) and saponified with NaOH. Then boron trifluoride-methanol solution was used to methyl-esterify the saponified fatty acids (Wijngaarden, 1967), which were then analyzed by gas chromatography (Agilent, 7820a). The data were initially sorted in Excel 2010. Then completely random analysis of variance was performed in SAS (version 8.2), and the LSD method was used to compare the significance of the differences.

#### 2.4.2. 16S rRNA gene PCR and sequencing

The 16S rRNA V3–V4 region of the eukaryotic ribosomal RNA gene was amplified by PCR (95°C for 2 min; followed by 27 cycles at 98°C for 10 s, 62°C for 30 s, and 68°C for 30 s; and a final extension at 68°C for 10 min) using primers 341F: CCTACGGGNGGCWGCAG and 806R: GGACTACHVGGGTATCTAAT, where the barcode was an eight-base sequence unique to each sample. PCR reactions were performed in triplicate 50 μL mixture containing 5 μL 10× KOD buffer, 5 μL 2.5 mM dNTPs, 1.5 μL of each primer (5 μM), 1 μL KOD polymerase, and 100 ng template DNA.

The 16S rRNA was sequenced with the Hiseq2500 PE250 platform (Guangzhou Gene Denovo Technology, Guangzhou, China; Caporaso et al., 2010).

#### 2.4.3. Number of operational taxonomic units and species classification

##### 2.4.3.1. Number of OTUs

We clustered effective tags into OTUs of ≥97% similarity using UPARSE pipeline (Edgar, 2013). The tag sequence with the highest abundance was selected as a representative sequence of the cluster. Between-groups Venn analysis was performed in R to identify unique and common OTUs. The multiple comparison for the OTUs analysis were applied using the LSD method (least significance difference test) analyzed by completely random analysis of variance in SAS (V8.2).

##### 2.4.3.2. Species classification

Only taxa with an abundance of greater than 1% were selected to classify species. The classification of microbial species includes seven grades: boundary, phylum, class, order, family, genus, and species. The two groups of samples were divided into species classification trees according to the distribution of each level. Assessments of the differences in dominant species in different samples and multiple sequence alignment were conducted with MUSCLE (V3.8.31; Edgar, 2004).

#### 2.4.4. Analysis of alpha diversity

Chao1, Simpson, and all other alpha diversity indices were calculated in QIIME (version 1.9.1; Caporaso et al., 2010). OTU rarefaction and rank abundance curves were plotted in QIIME. Between-groups comparisons of alpha index were calculated with Welch’s t test and the Wilcoxon rank test in R. Comparisons of alpha index among groups were performed with Tukey’s HSD test and the Kruskal-Wallis H test in R.

#### 2.4.5. Analysis of beta diversity

Beta diversity analysis was used to evaluate differences in samples in terms of species complexity. The beta diversity of both weighted and unweighted UniFrac was calculated with QIIME (version 1.9.1).

Cluster analysis was preceded by principal component analysis, which was performed to reduce the dimension of the original variables using the FactoMineR and ggplot2 packages in R (version 2.15.3).

Unweighted pair-group method with arithmetic means (UPGMA) clustering was performed as a type of hierarchical clustering to interpret the distance matrix using the average linkage and was performed in QIIME (version 1.9.1).

#### 2.4.6. Correlation analysis

The Pearson correlation coefficient between every level of bacteria and fatty acids were calculatedin R (V3.5.1). The network analysis was performed using igraph package in R.

## 3. Results

### 3.1. Fat and fatty acids

There was no significant difference in intramuscular fat content between the biceps femoris and longissimus dorsi of Tan lambs. According to the results for 37 fatty acids in the biceps femoris and longissimus dorsi of Tan lambs in the two feeding conditions, the content of 10 fatty acids was significantly higher in the biceps femoris of Tan lambs in the pen-ration group than the pasture group (*p* < 0.05), and the content of two fatty acids was significantly lower than in the pasture group (*p* < 0.05). Moreover, the content of 11 fatty acids was significantly higher in the longissimus dorsi of Tan lambs in the pen-ration group than in the pasture group (*p* < 0.05), and the content of five fatty acids was significantly lower than in the pasture group (*p* < 0.05; Supplementary Table 2). The fatty acids c18:3n3 and c20:5n3 were significantly higher in the biceps femoris and longissimus dorsi of the pasture group than the pen-ration group (*p* < 0.05). Different feeding patterns had no significant effect on the intramuscular fat content of the biceps femoris and longissimus dorsi of Tan lambs, but there was a significant effect on fatty acids composition.

### 3.2. Number of OTUs and species classification

#### 3.2.1. Number of OTUs

**Figure 1:**
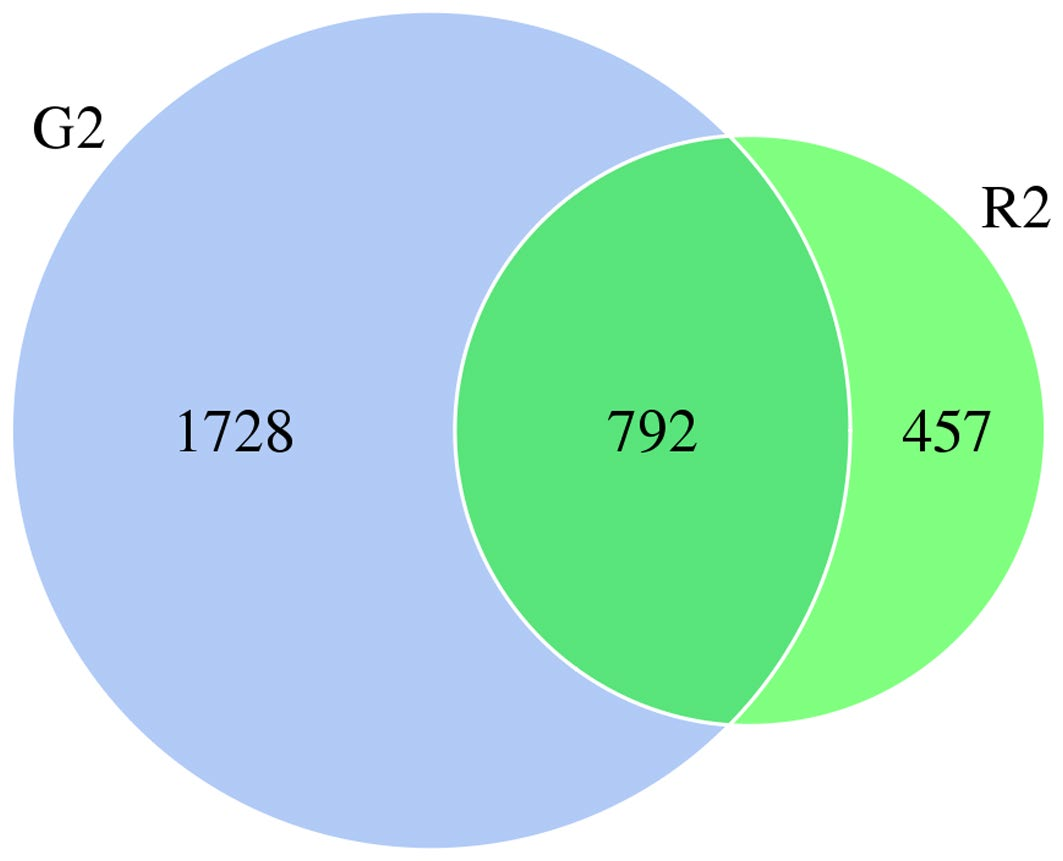
Venn diagram of microbial OTUs in the rumen fluid of two feeding groups (G2, pasture group; R2, pen-ration group) of Tan lambs.

When the microbial community of the two different habitats was analyzed, the distribution of some species was similar, and the distribution of others was specific to one or the other habitat to a certain extent. As shown in the Venn diagram in Figure 1, there were 2,520 OTUs of rumen bacteria in the pasture group and 1,249 in the pen-ration group. Of these OTUs, 792 were found in both groups. The number of OTUs of rumen bacteria was significantly higher in the pasture group than in the pen-ration group (Supplementary Table 3). Thus, the microbial diversity of rumen fluid was significantly higher in the pasture group than in the pen-ration group.

#### 3.2.2. Species classification

**Figure 2:**
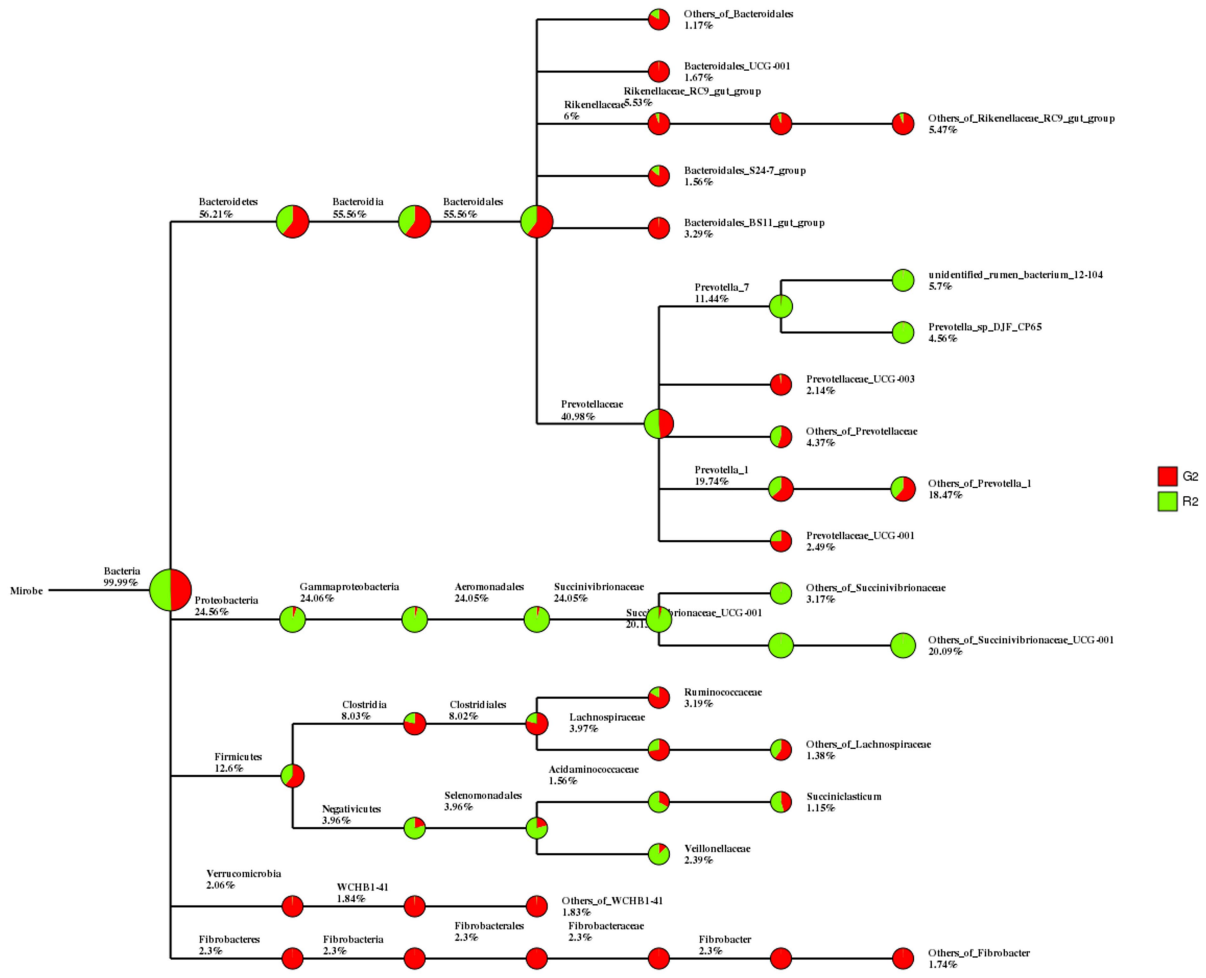
Classification tree for microbial groups in the rumen fluid of Tan sheep in two feeding groups: pasture group (G2, red) and pen-ration group (R2, green).

In the classification tree for rumen contents, Proteobacteria were found mostly in the pen-ration group, whereas almost all Verrucomicrobia and Fibrobacteres were in the pasture group (Figure 2).

### 3.3. Diversity of the rumen microbiome

#### 3.3.1. Alpha diversity

##### 3.3.1.1. OTU rarefaction curve and Shannon rarefaction curve

**Figure 3:**
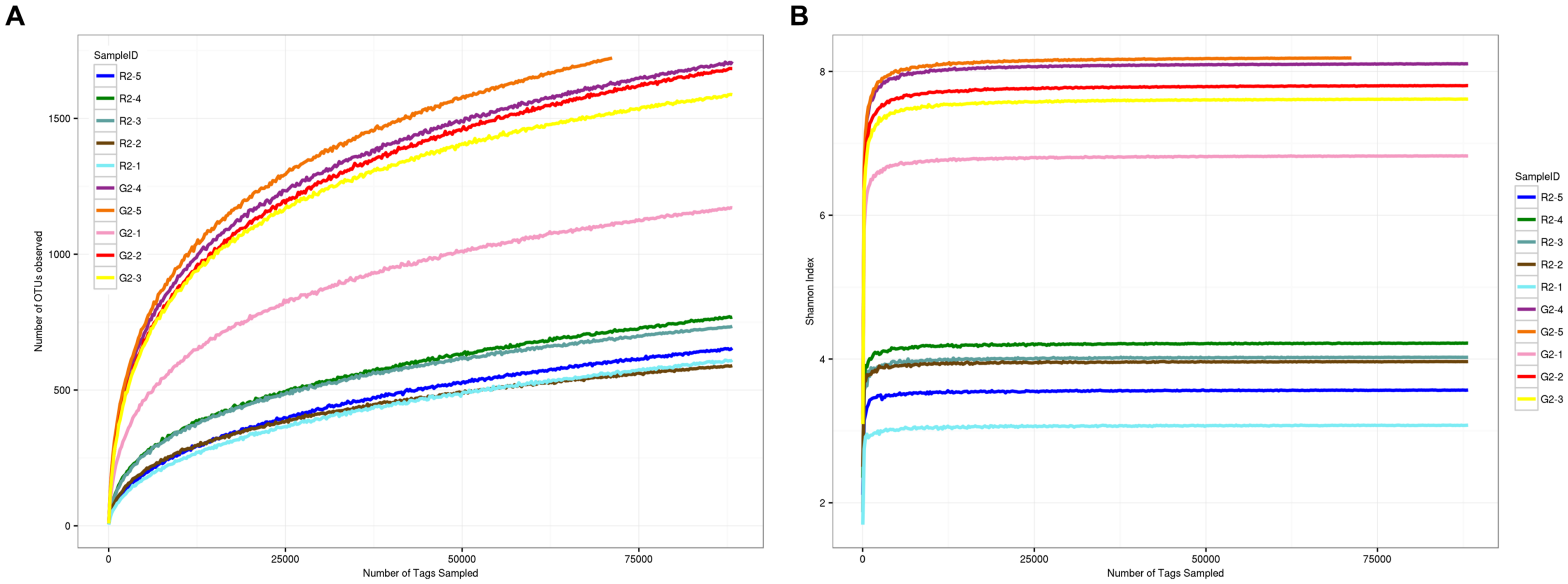
**(A)** OTU dilution curve for samples at a 0.03 distance. **(B)** Shannon dilution curve for samples at a 0.03 distance.

As shown in Figures 3A,B, when the sequence depth reached 75,000 reads, the two groups of curves tended to flatten, which indicates that the current sequence depth was sufficient to cover all species in the sample and for the analysis of sample microbial diversity.

##### 3.3.1.2. Analysis of alpha diversity

Alpha diversity, or the diversity in a particular habitat or ecosystem, can indicate the degree of isolation of the habitat based on the species. As shown in Supplementary Table 4, the sample coverage rate was greater than 99%, which indicates that the sample collection was sufficient to reflect the diversity of the rumen microbiome. The ACE index and Chao1 index were significantly higher in the pasture group than in the pen-ration group (*p* < 0.01), which indicates that the richness of the rumen microbiome was greater in the pasture group than in the pen-ration group. The Shannon index and Simpson index were also greater in the pasture group than in the pen-ration group, which indicates that the diversity of rumen bacteria was significantly higher in the pasture group than in the pen-ration group (*p* < 0.01).

#### 3.3.2. Beta diversity

##### 3.3.2.1. Beta diversity index

**Figure 4:**
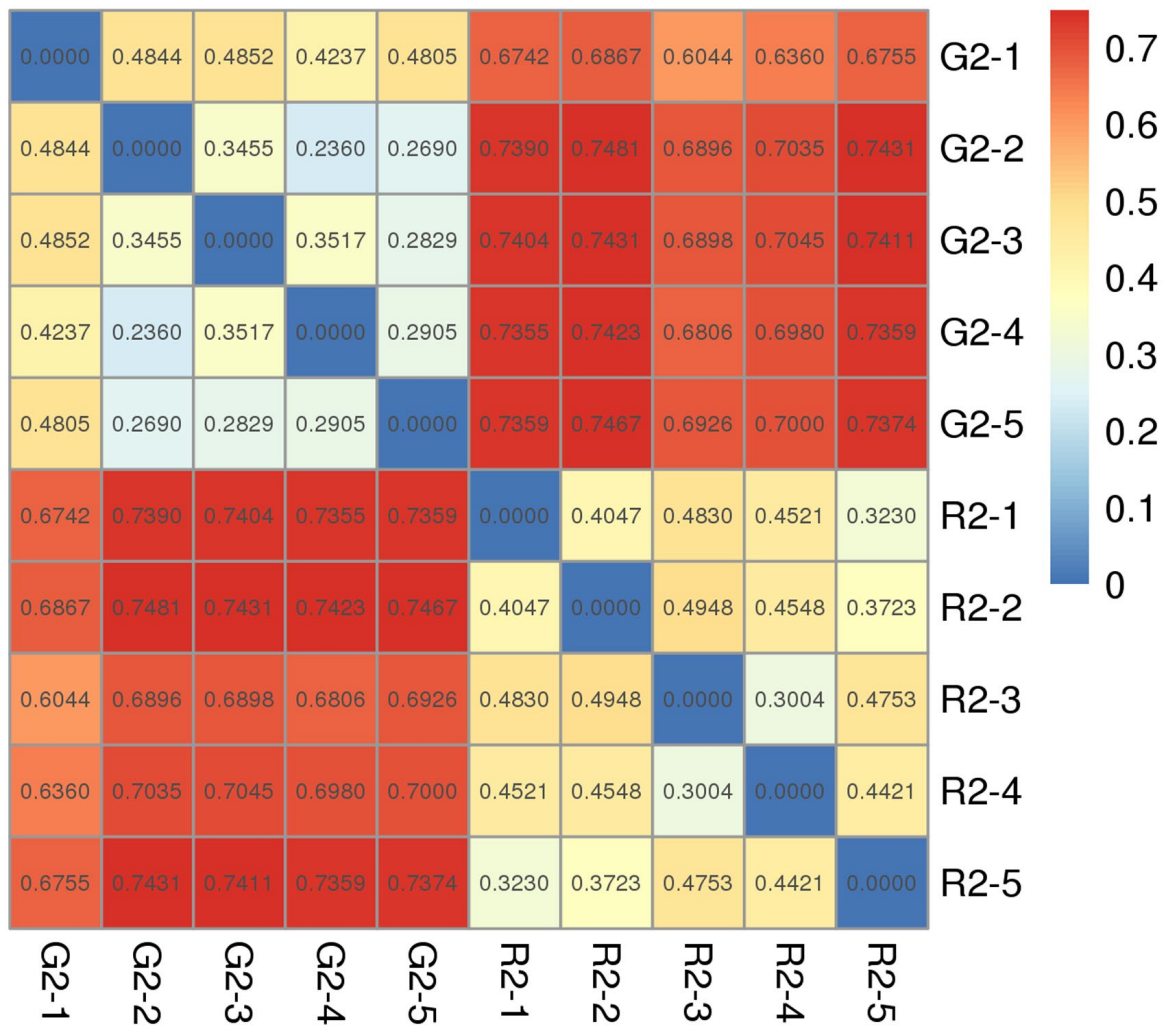
Unweighted Unifrac index heat map for rumen microbial samples from two different feeding groups of Tan lambs: pasture group (G2) and pen-ration group (R2).

The distance between samples in the pen-ration group was 0.3 to 0.4; thus, no significant difference in bacteria was detected among samples in this group. The control distance in the pasture group was 0.2 to 0.4, which indicates that bacteria were less homogeneous in the pasture group than in the pen-ration group (Figure 4). Regarding the presence or absence of species in the two groups, the distance value of the pasture group was higher than that of the pen-ration group, which indicates that the diversity of rumen bacteria was greater in the pasture group than in the pen-ration group. After we waited for abundance, the difference among microbiota in both groups was further reduced. Thus, the distance between samples was 0.0 to 0.1 in the pen-ration group and 0.1 to 0.3 in the pasture group.

##### 3.3.2.2. Principal component analysis

**Figure 5:**
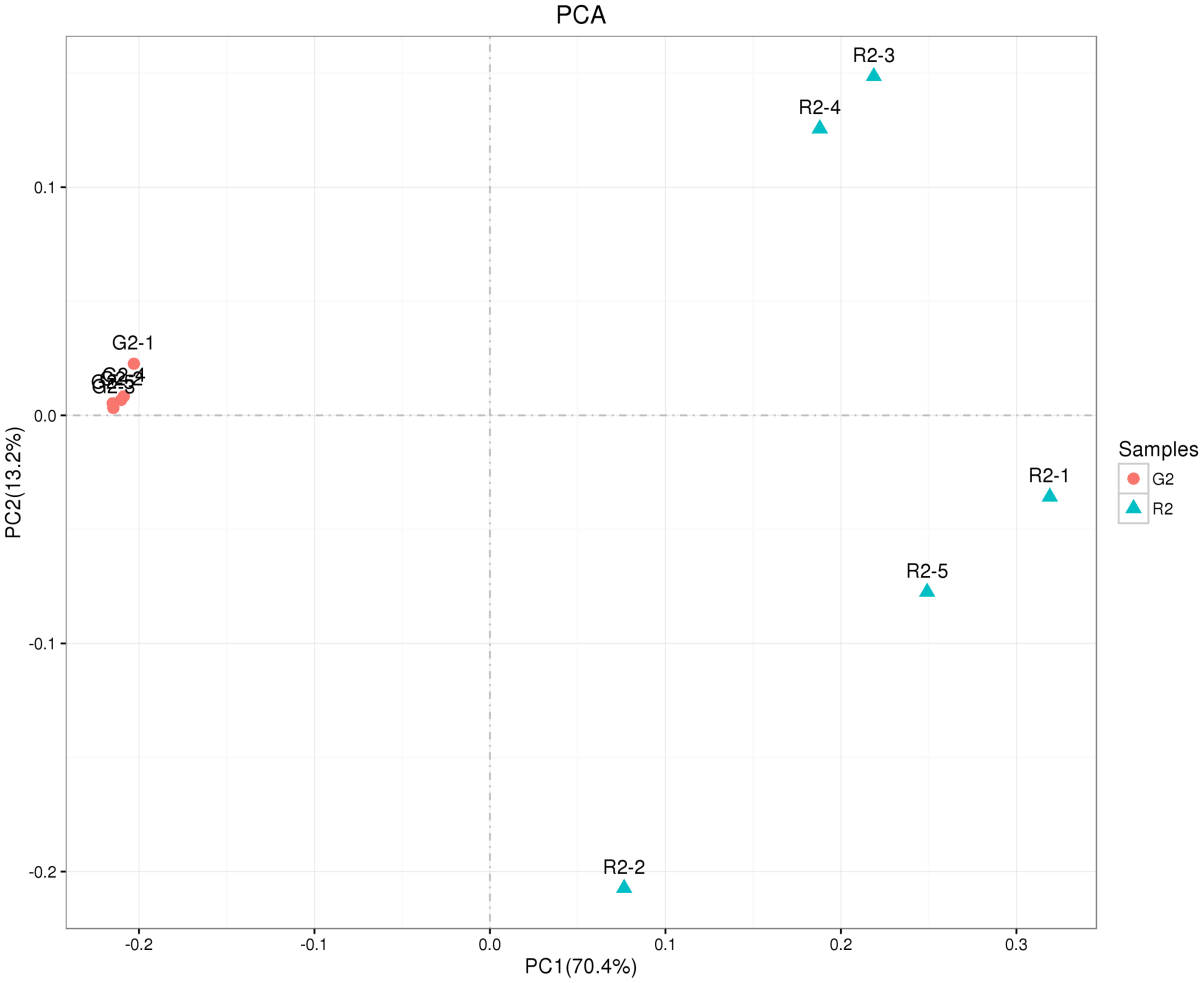
OTU PCA plot of the rumen microbial community in Tan lambs from two different feeding groups: pasture group (G2) and pen-ration group (R2).

As shown in Figure 5, the abscissa is the first principal component, and the ordinate is the second principal component. On the abscissa, the samples of the two feeding groups were completely separate and distinct, which indicates that the structure of the bacteria was different between the two groups. In the diagram for the whole cluster analysis, in the first principal factor (70.4%) and second principal factor (3.2%), the distribution of samples in the pen-ration group was concentrated, and the distribution of samples in the pasture group was dispersed; the two groups of samples were completely separate in the first principal factor. This shows that the uniformity of the pen-ration group was high, the uniformity of the pasture group was poor, and the difference between the two groups was obvious.

##### 3.3.2.3. UPGMA cluster analysis

**Figure 6:**
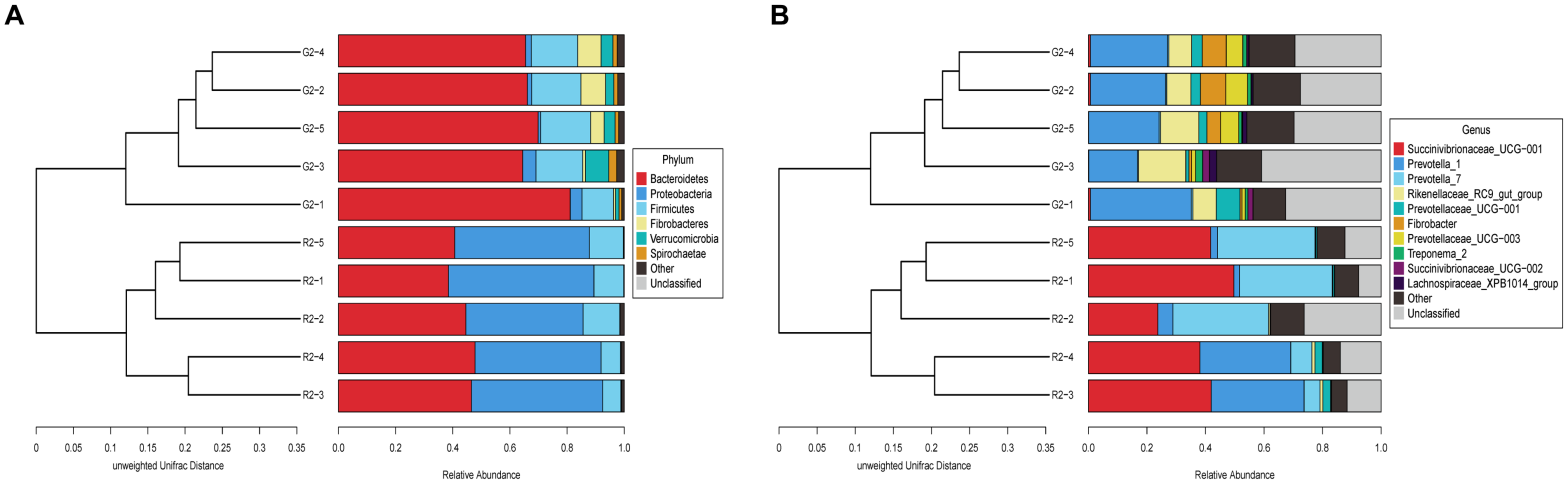
**(A)** UPGMA clustering tree at the phylum level for rumen microbes in two feeding groups of Tan lambs: pasture group (G2) and pen-ration group (R2). **(B)** UPGMA clustering tree at the genus level for rumen microbes in two feeding pattern of Tan lambs: pasture group (G2) and pen-ration group (R2).

At the phylum level in the UPGMA classification tree, the relative abundance of Bacteroidetes and Fibrobacteres was significantly higher in the pasture group than in the pen-ration group (Figure 6A), and these phyla were the dominant bacteria in the rumens of the pasture group of Tan lambs. Proteobacteria and Bacteroidetes were the dominant bacteria in the rumens of the pen-ration group. The relative abundance of Proteobacteria was significantly higher in the pen-ration group than in the pasture group (*p* < 0.01). At the genus level, the relative abundance of *Succinivibrionaceae_UCG-001* and *Prevotella_7* was significantly higher in the pen-ration group than in the pasture group (*p* < 0.01; Figure 6B), whereas the relative abundance of *Rikenellaceae_RC9_gut_group*, *Fibrobacter*, and *Treponema* was higher in the pasture group than in the pen-ration group.

### 3.4. Analysis of the structure of rumen bacteria

#### 3.4.1. Phylum level

Among the dominant phyla (Supplementary Table 5), the relative abundance of Bacteroidetes, Verrucomicrobia, and Proteobacteria was significantly higher in the pasture group than in the pen-ration group (*p* < 0.01), and the relative abundance of Firmicutes and Fibrobacteres was significantly higher in the pen-ration group than in the pasture group (*p* < 0.05).

#### 3.4.2. Genus level

With further refinement of the classification to the genus level, we identified 199 genera from the rumen communities of the two feeding groups, including 176 genera in the pasture group and 113 genera in the pen-ration group. Only 38 genera were identified with relatively high abundance, accounting for more than 92% of the total genera in each sample (Supplementary Table 6). The relative abundance of *Prevotella_7* and *Succinivibrionaceae_UCG-001* was significantly higher in the pen-ration group than in the pasture group (*p* < 0.01), and *Succinivibrionaceae_NA* was significantly higher in the pen-ration group than in the pasture group (*p* < 0.05). The relative abundance of *Prevotellaceae_UCG-003*, *Prevotellaceae_NK3B31_group*, *Rikenellaceae_RC9_gut_group*, *NA*, *Ruminococcus_1*, *Ruminococcus_2*, *Treponema_2*, *Christensenellaceae_R-7_group*, *Ruminococcus_1*, and *Butyrivibrio_2* was significantly higher in the pasture group than in the pen-ration group (*p* < 0.01). In addition, the relative abundance of *Fibrobacter*, *Succinivibrionaceae_UCG-002*, and *Succinivibrionaceae_NA* was significantly higher in the pasture group than in the pen-ration group (*p* < 0.05).

### 3.5. Correlation analysis

**Figure 7:**
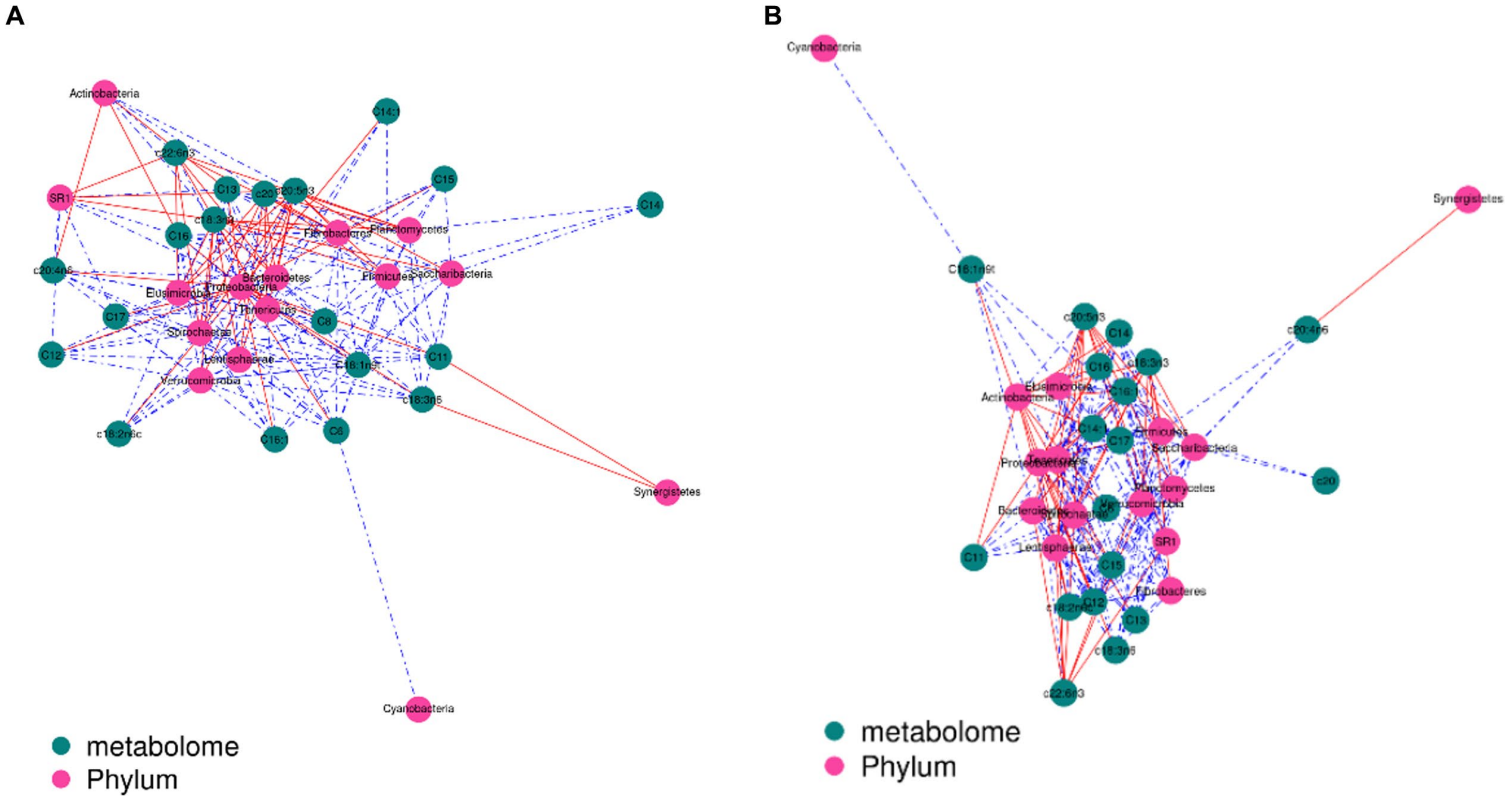
Correlation analysis between phylum level and fatty acids: blackish green, fatty acids; purplish red, phylum; red line, positive correlation; blue line, negative correlation. **(A)** Longissimus dorsi. **(B)** Biceps femoris.

**Figure 8:**
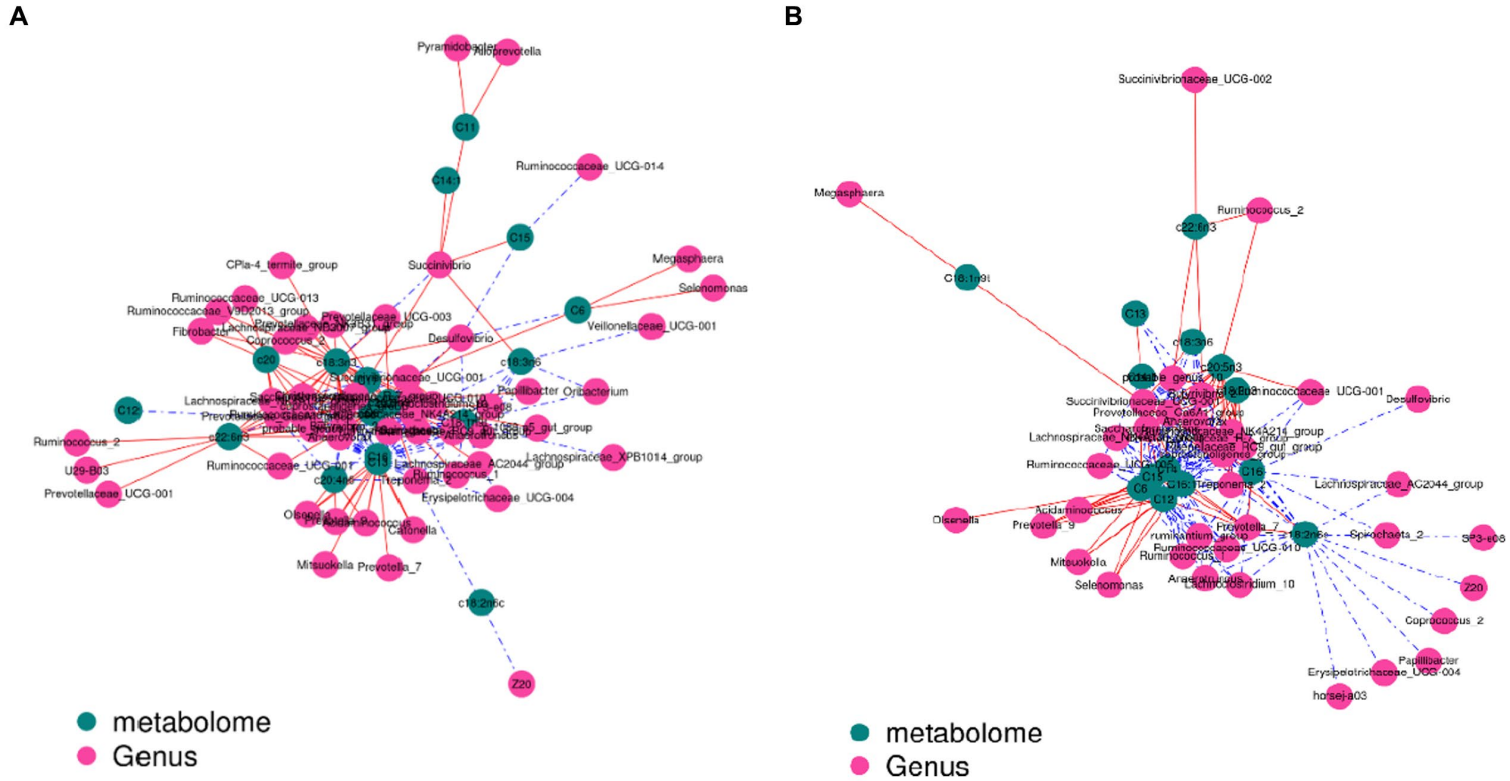
Correlation analysis between genus level and fatty acids: blackish green, fatty acids; purplish red, genus; red line, positive correlation; blue line, negative correlation. **(A)** Longissimus dorsi. **(B)** Biceps femoris.

The contents of c18:3n3 and c20:5n3 in the two muscles were positively correlated with the relative abundance of Tenericutes and Bacteroidetes (R^2^ > 0.75) and negatively correlated with Proteobacteria (R^2^ < −0.75) (Figure 7). The contents of c18:3n3 and c20:5n3 in the two muscles were positively correlated with *Christensenellaceae_R-7_group*, *Ruminococcaceae_NK4A214_group*, *Butyrivibrio_2*, *Saccharofermentans*, *Lachnospiraceae_NK4A136_group*, *Anaerovorax*, *coprostanoligenes_group*, *probable_genus_10*, *Ruminococcaceae_UCG-005*, and *Prevotellaceae_Ga6A1_group* (R^2^ > 0.75) and negatively correlated with *Succinivibrionaceae_UCG-001* (R^2^ < −0.75) (Figure 8). The correlations between c18:2n6c, C14, C6, C15, C12, C16:1, and C16 and the above-mentioned bacteria were the opposite.

## 4. Discussion

Dietary fatty acids consumed by sheep, like other ruminants, can undergo biohydrogenation resulting in high proportions of saturated FA in meat (Polan et al., 1964). Biohydrogenation is typically less extensive in sheep than cattle, and consequently, sheep meat can contain higher proportions of omega (n) − 3 polyunsaturated FA (PUFA), and PUFA biohydrogenation intermediates (PUFA-BHI) including conjugated linoleic acid (CLA) and trans-monounsaturated FAs (t-MUFA) (Carreño et al., 2019). Sheep meat is also noted for having characteristically higher contents of branched chain FA (BCFA). From a human health and wellness perspective, some SFA and trans-MUFA have been found to negatively affect blood lipid profiles, and are associated with increased risk of cardiovascular disease (CVD). On the other hand, n − 3 PUFA, BCFA and some PUFA-BHI may have many potential beneficial effects on human health and wellbeing. In particular, vaccenic acid (VA), rumenic acid (RA) and BCFA may have potential for protecting against cancer and inflammatory disorders among other human health benefits (Dilzer and Park, 2012; Calder, 2015). Holl et al. (2005) reported that the content of polyunsaturated fatty acids in fat samples from Hungarian Grey and Holstein Friesian bulls changed differently because of different diets. The intramuscular fat content is primarily determined by the number of preadipocytes, which is influenced by genetic and nutritional factors. As animals age, fat synthesis gradually increases while muscle mass decreases, leading to the deposition of intramuscular fat primarily in the late stages of animal maturity. Therefore, the two-month experimental period of this study may not fully capture the impacts of pasture-fed environment and pen-fed environment on fatty acid composition. In the future, it is advisable to extend the experimental period for in-depth research. In our experiments, there was no significant difference in intramuscular fat content, but there was a significant difference in fatty acids content: c18:3n3 and c20:5n3 were significantly higher in the biceps femoris and longissimus dorsi of the pasture group than the pen-ration group. Research has found that grazing can stimulate goats to have a higher intake of forage, thereby increasing the proportion of c18:3n3 (Thongruang and Paengkoum, 2019). Ebrahimi et al. (2014) reported that the reduction of SFA in intramuscular tissue of Boer kid goats obtained by feeding flaxseed oil was mainly due to the inhibitory effect of α-linolenic acid (c18:3n3) and/or its biohydrogenation products on *de novo* FA synthesis. Previous studies have indicated that c18:3n3 and c20:5n3 can serve as representative compounds in the muscle and fat tissues of grazing lambs, aiding in the differentiation of their geographical origin (Vasilev et al., 2020). Ekiz et al. (2013) also found differences in intramuscular fat and fatty acids content in 45 Kivircik lambs under different feeding conditions: Lambs of U-W-G (n = 12) were kept together with the main flock and grazed on pasture in the daytime, they and the pasture group had similar feeding conditions, and 18:3n3 and c20:5n3 were higher than for the other feeding patterns. In conclusion, different feeding patterns have no significant effect on the intramuscular fat content of Tan lambs but have a significant effect on fatty acids content, such that grazing is more conducive to the synthesis of 18:3n3 and c20:5n3, which are related to the biological hydrogenation of rumen microorganisms.

The rumen is the main digestive organ and supplier of energy in ruminants, with 70–85% of digestible substances and 50% of crude fibers in feed digested in the rumen (Zhou and Chen, 2010). Digestion depends on the complex rumen microbiome. Rumen microbial bacteria mainly include bacteria, anaerobic fungi, and protozoa (Xie et al., 2018), of which bacteria are the most abundant. In this study, indices of rumen bacterial richness (ACE index, Chao1 index) and diversity (Shannon index, Simpson index) were significantly lower in the pen-ration group than in the pasture group. This is consistent with previous research. For example, Grilli et al. (2016) found that a high-grain diet simplified and reduced the diversity index of the rumen microbiome. Moreover, Yáñez-Ruiz et al. (2010) found that the structure and composition of rumen microbial bacteria are related to diet type. In one study, there were fewer rumen bacteria in lambs fed a high-concentrate diet than in pasturing lambs, which shows that the high-concentrate diets was negatively correlated with the microbial composition of the rumen (Ji et al., 2016). In conclusion, pen rationing leads to lower bacterial diversity in the rumens of Tan lambs than pasturing.

The rumen is free of microorganisms at birth (Ziolecki and Briggs, 1961; Vi et al., 2004). Just 24 h after birth, facultative anaerobic bacteria are found in the rumen wall, and after 2 days, strict anaerobic microorganisms appear in the rumen. Before adulthood, the bacteria in the rumen undergo constant changes (Feng, 2004). Firmicutes, Bacteroidetes, and Proteobacteria are the dominant rumen bacteria (Qin et al., 2010; Singh et al., 2012; Li et al., 2018a). In this study, at the age of 2 months, the relative abundance of Bacteroidetes and Firmicutes in the rumen fluid of the pasture group reached more than 85%. The relative abundance of Bacteroidetes, Firmicutes, and Proteobacteria in the rumen fluid of the pen-ration group reached more than 99%. Moreover, the abundance of Proteobacteria was significantly higher in the pen-ration group than in the pasture group, whereas the abundance of Bacteroidetes and Firmicutes was significantly lower in the pen-ration group than in the pasture group. Thus, the two different feeding patterns significantly affected the abundance of dominant bacteria in the rumens of these Tan lambs.

Rumen bacteria primarily decompose cellulose and hemicellulose in roughage by enzymatic action (Zhang et al., 2018). Wang et al. (2010) reported that pasturing lambs showed cellulase activity on the day of their birth, and peaks in amylase and cellulase activity were detected at 42 days. The primary rumen microorganisms involved in the degradation of dietary fibers are *R. flavefaciens*, *R. albus*, *B. fibrisolvens*, and *F. succinogenes*, among others (Stewart et al., 1997; Stevenson and Weimer, 2009). Saro et al. (2014) found that the abundance of some cellulolytic bacteria and fungi was higher in the rumens of sheep fed a lower quality forage. Yang et al. (2015) showed that adding roughage to calf granules significantly increased the abundance of *R. albus*, *B. fibrisolvens*, *Ruminobacter amylophilus*, and *Lactobacillu*s in the rumen. In our experiments, pasturing significantly increased the abundance of *Fibrobacter*, *Ruminococcus*, *Butyrivibrio*, and *Pseudobutyrivibrio*, whereas pen feeding increased the abundance of *Prevotella_7* and *Succinivibrionaceae_UCG-001* in the rumens of Tan lambs.

Cao et al. (2016) found that protein-degrading and starch-degrading bacteria were more abundant in pen-fed yaks than in pasturing yaks. *Prevotella* is often associated with the degradation and utilization of starch, proteins, and polysaccharides in the rumen (Li et al., 2018b; Paz et al., 2018); however, according to Koike et al. (2003), *Prevotella* may be involved in fiber degradation in the rumens of sheep. Furthermore, Osborne and Dehority (1989) confirmed that *P. ruminicola* contributes to the degradation of the plant cell wall by acting synergistically with cellulolytic bacteria. In the current experiment, *Prevotella_1* and *Prevotella_7* were the dominant bacteria in the rumens of the pen-ration group. However, *Prevotella_7* was not a dominant bacterium in the pasture group and was significantly lower than in the pen-ration group. This difference in the genus composition of the two feeding groups is of great functional importance and means that *prevotella-1* and *prevotella-7* may participate in the degradation of different components of the ruminant diet.

## 5. Conclusion

The rumen bacterial community of Tan sheep is significantly influenced by pen-ration and pasture-fed conditions, leading to variations in fatty acid content in the muscle, which in turn affects the flavor and nutritional value of the meat to some extent. The diversity of rumen bacteria was significantly higher in a pasture group of Tan lambs than in a pen-ration group. The abundance of dominant bacteria, fiber-degrading bacteria, and starch-degrading bacteria in the rumens of the lambs was also significantly affected by the different feeding patterns, the abundance of dominant bacterial phyla and genera in pasture-fed sheep is significantly higher than that in pen-ration Tan sheep. In addition, the contents of c18:3n3 and c20:5n3 were significantly higher in the muscles of the Tan lambs in the grazing group, and the abundance of *Butyrivibrio_2*, *Pseudobutyrivibrio*, and *Succinivibrionaceae*, which are involved in rumen biohydrogenation, was higher in the pasture group. In summary, pasture-fed conditions have been shown to enhance the diversity of rumen bacterial community structure in Tan sheep, thereby increasing the nutritional value of their meat.

## Data availability statement

The data that support the findings of this study are available from the corresponding author upon reasonable request, and the sequencing data are available from NCBI. The BioProject number is PRJNA974932.

## Ethics statement

The animal study was approved by all experimental procedures and animal experiments were performed following the guidelines of the relevant Ethics Committee. This experimental procedure was approved by the Institutional Animal Care and Use Committee at Ningxia University (NXU1074901). The study was conducted in accordance with the local legislation and institutional requirements.

## Author contributions

LZ, WR and XX: conceptualization. YB and JZ: methodology. LZ and WR: software. LZ, YB, and YC: validation. LZ and WR: formal analysis, investigation. XX: resources. XX and WR: data curation. LZ: writing—original draft preparation, writing review and editing, visualization, supervision, and funding acquisition. XX: project administration. All authors contributed to the article and approved the submitted version.
