# Supplementary material for: Effects of different feeding patterns on the rumen bacterial community of tan lambs, based on high-throughput sequencing of 16S rRNA amplicons
Source: Front Microbiol. 2023 Oct 19;14:1228935. doi: 10.3389/fmicb.2023.1228935 (PMC10621797; doi:10.3389/fmicb.2023.1228935)
Supplement: Supplementary file 1 [file Table_1.DOCX]

TABLE 1.Composition and nutrient levels of the starting diet (dry matter basis) %.

| Ingredient | Content | Nutrient level | Content |
| --- | --- | --- | --- |
| Corn | 55 | Crude protein | 20.50 |
| Soybean meal | 30 | Ca | 0.73 |
| Wheat bran | 7 | P | 0.44 |
| Molasses | 5 | Neutral detergent fibre | 10.35 |
| CaHPO_4_ | 1.50 | Digestible energy (MJ/kg)^2^) | 11.60 |
| NaCl | 1 |  |  |
| Premix^1^) | 0.50 |  |  |
| Total | 100 |  |  |

1) Each kg of premix contains: vitamin A: 15 × 10^4^ IU, vitamin D_3_: 5 × 10^4^ IU, vitamin E: 20 × 10^4^ IU, niacin: 200 mg, pantothenic acid: 100 mg, biotin: 10 mg, Fe: 1600 mg, Cu: 250 mg, Mn: 2650 mg, Zn: 1000 mg, I: 15 mg, Se: 7.5 mg, Co: 7.5 mg.

2) Digestible energy(DE) was a calculated value; all others were measured values.

**TABLE 2** Intramuscular fat content and fatty acid composition of biceps femoris and longissimus dorsi (g·100 g^-1^)

| Item | Biceps femoris | | | Longissimus dorsi | | |
| --- | --- | --- | --- | --- | --- | --- |
|  | Pen-ration group | Pasture group | *p* | Pen-ration group | Pasture group | *p* |
| Fat (%) | 2.26 | 2.11 | 0.316 | 1.73 | 1.98 | 0.242 |
| C6 | 0.0138^A^ | 0.0007^B^ | 0.001 | 0.0039^a^ | 0.0006^b^ | 0.038 |
| C8 | 0.0015 | 0.0005 | 0.173 | 0.0008^a^ | 0.0002^b^ | 0.015 |
| C11 | 0.0010 | 0.0005 | 0.058 | 0.0056^A^ | 0.0003^B^ | 0.008 |
| C12 | 0.0117^A^ | 0.0004^B^ | 0.001 | 0.0102 | 0.0037 | 0.062 |
| C13 | 0.0016^A^ | 0.0002^B^ | 0.003 | 0.0014^A^ | 0.0003^B^ | 0.003 |
| C14 | 0.0975^A^ | 0.0418^B^ | 0.007 | 0.108 | 0.0590 | 0.101 |
| C14:1 | 0.0072^A^ | 0.0002^B^ | 0.005 | 0.0062^a^ | 0.0010^b^ | 0.035 |
| C15 | 0.0160^A^ | 0.0012^B^ | 0.001 | 0.0138 | 0.0083 | 0.108 |
| C16 | 0.9814^A^ | 0.3030^B^ | 0.000 | 1.2000^A^ | 0.3370^B^ | 0.002 |
| C16:1 | 0.0397^A^ | 0.0133^B^ | 0.000 | 0.0493^a^ | 0.0206^b^ | 0.027 |
| C17 | 0.0328 | 0.0278 | 0.678 | 0.0356^a^ | 0.0212^b^ | 0.018 |
| C18:1n9t | 0.0383 | 0.0198 | 0.135 | 0.0459^A^ | 0.0178^B^ | 0.003 |
| c18:2n6c | 0.1402^A^ | 0.1050^B^ | 0.001 | 0.1265 | 0.1090 | 0.166 |
| c20 | 0.0012 | 0.0007 | 0.229 | 0.0004^b^ | 0.0014^a^ | 0.032 |
| c18:3n6 | 0.0042^a^ | 0.0016^b^ | 0.030 | 0.0035^a^ | 0.0015^b^ | 0.016 |
| c18:3n3 | 0.0101^B^ | 0.0259^A^ | 0.003 | 0.0089^B^ | 0.0233^A^ | 0.000 |
| c20:4n6 | 0.0630 | 0.0497 | 0.078 | 0.0597^a^ | 0.0431^b^ | 0.030 |
| c20:5n3 | 0.0026^B^ | 0.0193^A^ | 0.000 | 0.0019^B^ | 0.0154^A^ | 0.000 |
| c22:6n3 | 0.0034 | 0.0102 | 0.121 | 0.0009^B^ | 0.0050^A^ | 0.009 |

In the same row, values with different lowercase superscripts are significantly different (*p* < 0.05). For values with different capital superscripts, the differences are highly significant (*p* < 0.01).

**TABLE 3** Number of OTUs in the rumen samples of Tan lambs in the two feeding groups

| Item | Pasture group | | | Pen-ration group | | *p* |
| --- | --- | --- | --- | --- | --- | --- |
|  | Sample | | OTUs | Sample | OTUs |  |
|  | G2-1 | 1199 | | R2-1 | 647 |  |
|  | G2-2 | 1730 | | R2-2 | 593 |  |
|  | G2-3 | 1634 | | R2-3 | 737 |  |
|  | G2-4 | 1770 | | R2-4 | 808 |  |
|  | G2-5 | 1723 | | R2-5 | 685 |  |
| Average | 1611.2^A^ | | | 694^B^ | | 0.001 |

In the same row, values with different lowercase superscripts are significantly different (*p* < 0.05). For values with different capital superscripts, the differences are highly significant (*p* < 0.01).

**TABLE 4** Diversity indices of rumen samples from two feeding groups of Tan lambs: pasture group and pen-ration group

| Items | Pasture group | Pen-ration group | | *p* |
| --- | --- | --- | --- | --- |
| **Richness index** |  | |  |  |
| ACE index | 1913.600^A^ | 970.900^B^ | | 0.001 |
| Chao1 index | 1910.265^A^ | 951.180^B^ | | 0.001 |
| **Diversity index** |  | |  |  |
| Shannon index | 7.709^A^ | 3.770^B^ | | 0.000 |
| Simpson index | 0.985^A^ | 0.785^B^ | | 0.001 |
| Coverage | 0.998^a^ | 0.996^b^ | | 0.069 |

In the same row, values with different small-letter superscripts are significantly different (*p* < 0.05). For values with different capital-letter superscripts, the differences are highly significant (*p* < 0.01).

**TABLE 5** Effects of two different feeding patterns on the percentages of phyla in the rumen bacteria of Tan lambs

| Phylum | Pasture group | Pen-ration group | *p* |
| --- | --- | --- | --- |
| Bacteroidetes | 69.371^A^ | 43.600^B^ | 0.001 |
| Firmicutes | 15.656^a^ | 9.669^b^ | 0.010 |
| Fibrobacteres | 4.627^a^ | 0.007^b^ | 0.024 |
| Verrucomicrobia | 4.073^A^ | 0.030^B^ | 0.007 |
| Proteobacteria | 2.644^A^ | 45.795^B^ | 0.000 |
| Spirochaetae | 1.564^A^ | 0.096^B^ | 0.002 |
| Cyanobacteria | 0.669 | 0.573 | 0.739 |
| Lentisphaerae | 0.433^A^ | 0.001^B^ | 0.004 |
| Planctomycetes | 0.319^a^ | 0.000^b^ | 0.016 |
| SR1 | 0.169^a^ | 0.000^b^ | 0.039 |
| Tenericutes | 0.137^A^ | 0.001^B^ | 0.000 |
| Saccharibacteria | 0.097^a^ | 0.021^b^ | 0.024 |
| Elusimicrobia | 0.093^A^ | 0.018^B^ | 0.009 |
| Synergistetes | 0.078 | 0.108 | 0.412 |
| Bacteria_NA | 0.030 | 0.016 | 0.071 |
| Actinobacteria | 0.026 | 0.065 | 0.076 |
| Euryarchaeota | 0.010^A^ | 0.000^B^ | 0.001 |
| Armatimonadetes | 0.002^a^ | 0.000^b^ | 0.012 |
| WA-aaa01f12 | 0.001 | 0.000 | 0.060 |
| Chloroflexi | 0.001 | 0.000 | 0.245 |
| Fusobacteria | 0.001 | 0.000 | 0.621 |

In the same row, values with different lowercase superscripts are significantly different (*p* < 0.05). For values with different capital superscripts, the differences are highly significant (*p* < 0.01).

**TABLE 6** Effects of two different feeding patterns on the percentages of genera in the rumen bacteria of Tan lambs

| Genus | Pasture group | Pen-ration group | *p* |
| --- | --- | --- | --- |
| *Prevotella_1* | 25.439 | 14.428 | 0.159 |
| *Prevotella_7* | 0.432^B^ | 22.054^A^ | 0.006 |
| *Prevotellaceae_NA* | 4.846 | 3.827 | 0.671 |
| *Prevotellaceae_UCG-003* | 4.350^A^ | 0.077^B^ | 0.007 |
| *Prevotellaceae_UCG-001* | 3.756 | 1.252 | 0.066 |
| *Prevotellaceae_NK3B31_group* | 0.859^A^ | 0.001^B^ | 0.004 |
| *Rikenellaceae_RC9_gut_group* | 10.664^A^ | 0.621^B^ | 0.001 |
| *Rikenellaceae_NA* | 0.400^a^ | 0.000^b^ | 0.021 |
| *NA* | 8.907^A^ | 1.049^B^ | 0.002 |
| *Bacteroidales_BS11_gut_group_NA* | 6.696^A^ | 0.051^B^ | 0.000 |
| *Bacteroidales_UCG-001_NA* | 3.349^A^ | 0.024^B^ | 0.002 |
| *Bacteroidales_S24-7_group_NA* | 2.682 | 0.443 | 0.113 |
| *Bacteroidales_RF16_group_NA* | 1.501^A^ | 0.112^B^ | 0.000 |
| *Fibrobacter* | 4.627^a^ | 0.007^b^ | 0.016 |
| *Lachnospiraceae_NA* | 1.687 | 1.087 | 0.057 |
| *Lachnospiraceae_XPB1014_group* | 0.991^a^ | 0.002^b^ | 0.034 |
| *Lachnospiraceae_NK4A136_group* | 0.324^A^ | 0.025^B^ | 0.001 |
| *Treponema_2* | 1.388^A^ | 0.068^B^ | 0.001 |
| *Succiniclasticum* | 1.057 | 1.241 | 0.332 |
| *Succinivibrionaceae_UCG-001* | 0.455^B^ | 39.047^A^ | 0.000 |
| *Succinivibrionaceae_UCG-002* | 0.965^a^ | 0.003^b^ | 0.046 |
| *Succinivibrionaceae_NA* | 0.099^b^ | 6.378^a^ | 0.036 |
| *Christensenellaceae_R-7_group* | 1.083^A^ | 0.029^B^ | 0.000 |
| *Christensenellaceae_NA* | 0.014^A^ | 0.000^B^ | 0.007 |
| *Erysipelotrichaceae_UCG-004* | 0.805^a^ | 0.128^b^ | 0.013 |
| *SP3-e08* | 0.509^A^ | 0.000^B^ | 0.006 |
| *Ruminococcus_1* | 0.326^A^ | 0.079^B^ | 0.001 |
| *Ruminococcus_2* | 0.188^a^ | 0.015^b^ | 0.011 |
| *Ruminococcaceae_UCG-014* | 0.940 | 0.612 | 0.247 |
| *Ruminococcaceae_NA* | 0.782^A^ | 0.137^B^ | 0.001 |
| *Ruminococcaceae_UCG-010* | 0.606^A^ | 0.002^B^ | 0.001 |
| *Ruminococcaceae_UCG-002* | 0.573^a^ | 0.115^b^ | 0.012 |
| *Ruminobacter* | 0.325 | 0.031 | 0.273 |
| *Saccharofermentans* | 0.354^A^ | 0.000^B^ | 0.001 |
| *Butyrivibrio_2* | 0.331^A^ | 0.027^B^ | 0.000 |
| *Pseudobutyrivibrio* | 0.043^A^ | 0.003^B^ | 0.000 |

In the same row, values with different lowercase superscripts are significantly different (*p* < 0.05). For values with different capital superscripts, the differences are highly significant (*p* < 0.01).
